# Nitrate Accumulation and Expression Patterns of Genes Involved in Nitrate Transport and Assimilation in Spinach

**DOI:** 10.3390/molecules23092231

**Published:** 2018-09-02

**Authors:** Xiaoli Wang, Xiaofeng Cai, Chenxi Xu, Shui Wang, Shaojun Dai, Quanhua Wang

**Affiliations:** Development and Collaborative Innovation Center of Plant Germplasm Resources, Shanghai Engineering Research Center of Plant Germplasm Resources, College of Life and Environment Science, Shanghai Normal University, Shanghai 200234, China; cxf0012@163.com (X.C.); chenxixu@shnu.edu.cn (C.X.); shuiwang@shnu.edu.cn (S.W.); daishaojun@shnu.edu.cn (S.D.)

**Keywords:** *Spinacia oleracea*, nitrate accumulation, *NRT*, nitrate reductase

## Abstract

Excessive accumulation of nitrate in spinach is not only harmful to human beings, but also limits the efficiency of nitrogen usage. However, the underlying mechanism of nitrate accumulation in plants remains unclear. This study analyzed the physiological and molecular characteristics of nitrate uptake and assimilation in the spinach varieties with high or low nitrate accumulation. Our results showed that the variety of spinach with a high nitrate content (So18) had higher nitrate uptake compared to the variety with a low nitrate content (So10). However, the nitrate reductase activities of both varieties were similar, which suggests that the differential capacity to uptake and transport nitrate may account for the differences in nitrate accumulation. The quantitative PCR analysis showed that there was a higher level of expression of spinach nitrate transporter *(SoNRT)* genes in So18 compared to those in So10. Based on the function of Arabidopsis homologs *AtNRTs*, the role of spinach *SoNRTs* in nitrate accumulation is discussed. It is concluded that further work focusing on the expression of *SoNRTs* (especially for *SoNRT1.4*, *SoNRT1.5* and *SoNRT1.3*) may help us to elucidate the molecular mechanism of nitrate accumulation in spinach.

## 1. Introduction

Nitrate is a common chemical compound in Nature, widely found in soils, waters, and foods. Nitrate itself is relatively non-toxic, but its metabolites may produce a number of health effects, such as infantile methaemoglobinaemia, carcinogenesis and possibly even teratogenesis [1,2]. Vegetables are the major source of the daily intake of nitrate by human beings [3]. Although more recent work has shown that the potential health concerns of nitrate were somewhat exaggerated, nitrate content as an important determinant of the quality of vegetables draws attention around the world. Moreover, it is well known that the majority of the absorbed nitrate is stored in the vacuoles of both roots and shoots. The remobilization and re-utilization of this nitrate is a big challenge for increasing nitrogen use efficiency in vegetables [4,5]. Thus, the suppression of nitrate content in vegetables is a major concern also in terms of potential health hazards and its potential limitations in improving nitrogen use efficiency.

Understanding the mechanism of nitrate accumulation metabolism is a prerequisite for reducing nitrate content in vegetables. It is known that the equilibrium of nitrate concentration in vegetables depends mainly on the nitrate uptake, translocation, assimilation and storage. Factors that influence these process can affect nitrate concentration in vegetables, such as plant genetic factors, environmental factors (photoperiod, CO_2_, temperature, irrigation, etc.) and agricultural factors (nitrogen doses and chemical forms, availability of other nutrients, etc.) [6]. Many studies have been carried out, which focused on reducing nitrate content in vegetables, such as the optimization of the application of nitrogen fertilizers [7,8,9,10], rational application of nitrification inhibitors (3,4-dimethylpyrazole phosphate and dicyandiamide) [11], exogenous salicylic acid [12] and iodine fertilization [13], growing plants under proper light intensity [14], CO_2_ concentrations [15] and organic cropping systems [16]. However, up to now, little is known about the molecular mechanisms of nitrate accumulation in plants. 

Understanding the plant nitrate transporter and nitrate reductase (NR) enzymes provides us with clues to investigate the mechanism of nitrate accumulation in spinach. Plant nitrate uptake from soil depends on two systems: a low-affinity transporter system and a high-affinity transporter system. These involve two families of proteins, which are named nitrate transporter 1 (NRT1)/peptide transporter (PTR) and nitrate transporter 2 (NRT2), respectively [17,18]. Fifty-three putative NRT1/PTR (also known as NPF*)* genes were predicted in the genome of *Arabidopsis thaliana* and 10 were characterized as nitrate transporters [19]. Many NPF family members in other plant species, such as *Populus trichocarpa*, *Cucumis sativus* and *Oryza sativa*, were also identified [20,21,22]*.* Some of them were found to play an important role in nitrate accumulation in plants. For example, Arabidopsis NRT1.4 is a low-affinity nitrate transporter, which is dominantly expressed in the leaf petioles. The nitrate content of the petiole in *nrt1.4* mutants was reduced to 50–64% of the wild-type level [23]. Arabidopsis NPF2.3 is a member of the nitrate excretion transporter (NAXT) sub-group of the NRT1/PTR family (NPF). Both root-to-shoot nitrate translocation and nitrate content in shoots were reduced under saline stress in *npf2.3* mutants [24]. Expression of the *Cucumis sativus NRT1.7* in *Arabidopsis nrt1.7-2* mutants resulted in reduced nitrate content and larger leaf size [25]. Zhao et al. found that the expression of *BnNRT2* in root, leaves and stem in the variety with a high nitrate content was higher than that in the variety of Chinese cabbage with low nitrate *(Brassica campestris* ssp*. Chinensis* (L.) *Makino*) [26]. In maize, *ZmNrt2.1* may be related to maize nitrate accumulation, as its expression in response to nitrate availability was similar to that of the nitrate influx in roots [27]. All these findings have suggested that *NRT* genes may play an important role in regulation of the nitrate level in plants. However, up to now, little is known about the spinach *NRT* genes and their roles in nitrate accumulation remain unclear. 

Nitrate reductase (NR) is a key regulatory enzyme in the NO_3_^-^ assimilation pathway, which reduces NO_3_^−^ to NO_2_^−^. After this, the NO_2_^−^ was reduced to NH_4_^+^, while the NH_4_^+^ is incorporated into glutamine via glutamine synthetase (GS). It has been suggested that the high NR activity is beneficial for nitrate reduction, which subsequently decreases nitrate accumulation in plants [28]. The expression of the putative nitrate reductase genes (*NIA1* and *NIA2*) were significantly higher in the genotype of pakchoi with a low nitrate content compared to those that had a higher level of accumulated nitrate [29]. Moreover, overexpression of nitrate reductase genes in lettuce [30] and potato [31] was proven to be helpful in reducing the nitrate content of plants. GS, which is a key enzyme involved in ammonium assimilation, may also function in reducing the nitrate content in plants. However, few reports have focused on the relationship between GS and nitrate accumulation in plants. In summary, although the molecular physiology of nitrate dynamics is well understood in model systems, their attention was mainly devoted to enhancing crop production. Little attention has been paid to the nutritional quality traits determining nitrate accumulation in plants. Thus, the molecular mechanism of nitrate accumulation in plants needs to be investigated. 

Spinach (*Spinacia oleracea* L.) is an edible flowering plant cultivated as one of the most popular vegetables all over the world. It is identified as a good source of vitamins A, C and E, folic acid, minerals (USDA Nutrient Database; http://ndb.nal.usda.gov/ndb/search/list) and other bioactive compounds such as phenolics, carotenoids, glycoglycerol lipids, and lipoic acid. However, high levels of accumulated nitrate have often been found in spinach which may affect human health [2,32,33]. The FAO and WHO food commissions report that the average daily nitrate intake of a 60 kg person should be 220–240 mg. The European Union Food Commission States established an acceptable daily intake (ADI) for nitrate as 0–3.7 mg·kg^−1^ of body weight and set the maximum limits of nitrogen accumulation in spinach as 3000 and 2500 mg·kg^−1^ for winter and spring crops, respectively [34]. According to this, most of the spinach nitrate contents reported by various countries, such as Japan [32], France [35], New Zealand [36], Swedish [37], UK [38], Turkey [39], Korea [40] and China [41] were close to or exceeded to this standard. Thus, there is an urgent need to reduce nitrate accumulation in spinach. In this study, we compared the physiological and molecular characteristics of nitrate transport and assimilation between two spinach varieties. We aim to explore the main factors that contribute to nitrate accumulation in spinach and screen the potential genes to be targeted in reducing spinach nitrate content.

## 2. Results

### 2.1. Fresh Weight and Nitrate Content

**Figure 1:**
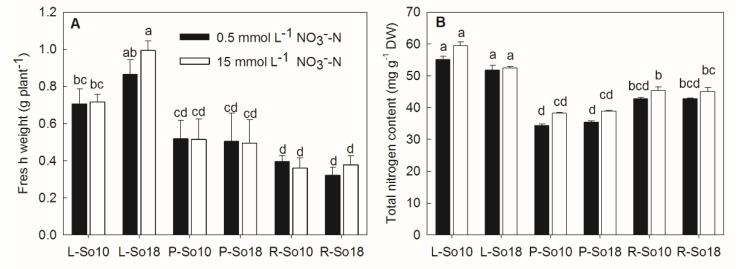
Fresh weights (**A**) and total nitrogen contents (**B**) of two spinach varieties (S10 and S18) after 12 h of nitrate treatment. L-So10, the leaf blades of S10; L-So18, the leaf blades of So18; P-So10, the petioles of So10; P-So18, the petioles of So18; R-So10, the roots of So10; and R-So18, the roots of So18. Different letters in columns indicate significant difference between the mean values at *p* < 0.05 by the LSD test (*n* = 3). Values are provided as mean ± SD.

The applied nitrate concentration did not have significant effects on the fresh weight and total nitrogen content of spinach (Figure 1). Significant differences in the tissue fresh weights between two varieties were only observed on leaf blades, as the fresh weight of leaf blade, of So18 was greater than that of So10 (Figure 1A). No significant differences in total nitrogen content were found between the two varieties (Figure 1B).

**Figure 2:**
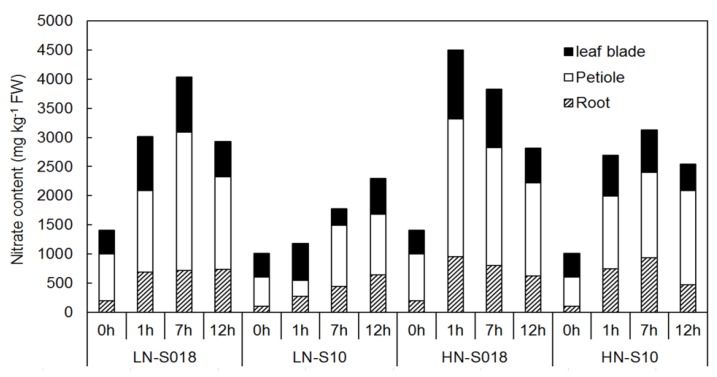
Nitrate contents in tissues of two spinach varieties under low (0.5 mmol·L^−1^) and high (15 mmol·L^−1^) nitrate treatments. LN-So18, So18 treated with low nitrate concentration; LN-So10, So10 treated with low nitrate concentration; HN-So18, So18 treated with high nitrate concentration; and HN-So10, So10 treated with high nitrate concentration.

The nitrate contents generally differed between the two spinach varieties and two nitrate concentration treatments (Figure 2). The nitrate contents in the whole plant of So18 were significantly greater than those in So10 at each treatment time, irrespective of the applied nitrate concentration. Among all treatments, the nitrate contents in leaf petioles were significantly greater than roots and leaf blades, except for the leaf petioles under low nitrate treatment at 1 h. Applying a greater nitrate concentration generally increased the whole plant nitrate contents for both varieties, except for So18 at 7 h and 12 h.

### 2.2. ^15^NO_3_^−^-N Uptake Rate

**Figure 3:**
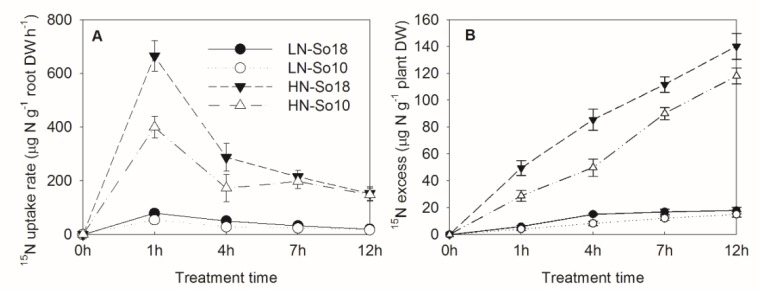
^15^NO_3_^−^-N uptake rate (**A**) and ^15^NO_3_^−^-N excess (net NO_3_^−^-N uptake amount) (**B**) of two spinach varieties under low (0.5 mmol·L^−1^) and high (15 mmol·L^−1^) nitrate treatments. Values are provided as mean ± SD, *n* = 3. LN-So18, So18 treated with low nitrate concentration; LN-So10, So10 treated with low nitrate concentration; HN-So18, So18 treated with high nitrate concentration; and HN-So10, So10 treated with high nitrate concentration.

As nitrate uptake by roots is the main source of nitrate accumulation in plants, we further analyzed the ^15^NO_3_^−^-N uptake rate and net ^15^NO_3_^−^-N uptake amount (^15^NO_3_^−^-N excess) and compared these values in the two varieties using isotopic labeling experiment. As shown in Figure 3, the ^15^NO_3_^−^-N uptake rate and ^15^NO_3_^−^-N excess of So18 was generally higher than those of So10 under both low and high nitrate conditions (*p* < 0.05).

The differences of ^15^NO_3_^−^-N uptake rates and ^15^NO_3_^−^-N excess between two varieties were significant at 1–4 h and 1–12 h (except those treated with a low nitrate concentration for 1 h), respectively. The So18 ^15^NO_3_^−^-N uptake amount was higher by 18.9% (low nitrate treatment) and 18.7% (high nitrate treatment) compared to So10 within 12 h, which suggested that So18 has a greater capacity for nitrate uptake from nutrient solution.

### 2.3. N Assimilation Related-Enzymes Activities

**Figure 4:**
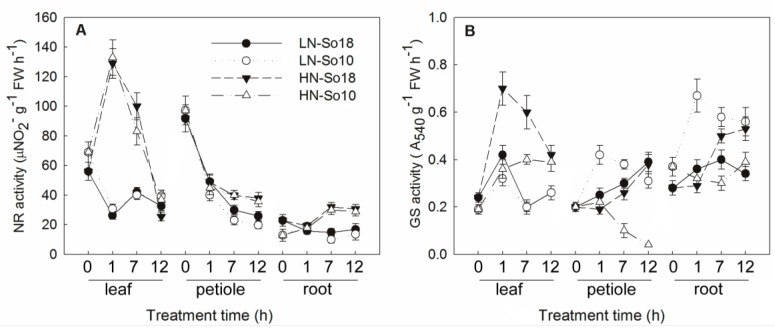
The activities of nitrate reductase (NR) (**A**) and glutamine synthetase (GS) (**B**) in shoots of two spinach varieties under low (0.5 mmol·L^−1^) and high nitrate (15 mmol·L^−1^) treatments. Values are represented as mean ± SD, *n* = 3. LN-So18, So18 treated with low nitrate concentration; LN-So10, So10 treated with low nitrate concentration; HN-So18, So18 treated with high nitrate concentration; and HN-So10, So10 treated with high nitrate concentration.

NR and GS are key enzymes involved in nitrate assimilation pathway, which play important roles in nitrate accumulation in spinach. The activities of two N assimilation related enzymes were analyzed in two spinach varieties under two different nitrate treatments. As shown in Figure 4, under low nitrate concentration treatment, the shoot NR activities of both two varieties decreased, while there was only a small change in the root NR activities. Under high nitrate concentration treatment, the dynamics of NR activities in petioles and roots were generally similar to those under low nitrate treatment. The NR activities in the leaf blade were the only exception as they first increased at 1 h and then decreased at 12 h. Under both low and high nitrate treatments, NR activities did not differ significantly between two varieties among the three tissues.

The dynamics of GS activities were more complex than those of NR. Generally, leaf blade GS activities of So18 were higher than those of So10 under both nitrate treatments. In petioles, the GS activities of two spinach varieties showed opposite trends in proportion to treatment time under both nitrate treatments. The petiole GS activities of So10 first increased at 1 h and were higher than those of So18 at 1 h, before decreasing at 12 h and becoming lower than those of So18. Under low nitrate conditions, root GS activities first increased then decreased at 12 h for both varieties, while this was reversed under high nitrate conditions. The So10 GS activities were generally higher than those of So18 under low nitrate conditions, while under high nitrate treatment, they did not differ from those of So18 at 1 h and were lower than those of So18 at 12 h. 

### 2.4. Expression Analysis of Nitrate Transport and Assimilation Related Genes

To demonstrate a correlation between the gene expression level and N concentration as well as its subsequent effect on nitrate accumulation, a qRT-PCR assay was performed on three spinach tissues (leaf blade, leaf petiole and root) of 12 *SoNRTs*, two *SoGLNs* and one *SoNIA* in two spinach varieties under two nitrate concentration treatments (Table S1). They identified 12 *SoNRTs*, which included: two homologs of *AtNRT1.1* (*SoNRT1.1a* and *SoNRT1.1b*), one homolog of *AtNRT 1.2* (*SoNRT1.*2), one homolog of *AtNRT 1.3* (*SoNRT1.3*), one homolog of *AtNRT1.4* (*SoNRT1.4*), one homolog of *AtNRT1.5* (*SoNRT1.5*), one putative spinach homolog of *AtNRT1.6* and *AtNRT1.7* (*SoNRT1.6*), two homologs of *AtNRT1.8* (*SoNRT1.8a* and *SoNRT1.8b*), two homologs of A*tNRT1.9* (*SoNRT1.9a* and *SoNRT1.9b*) and one homolog of *AtNRT2.1* (*SoNRT2*). All the tested genes were shown to be expressed in all spinach tissues.

**Figure 5:**
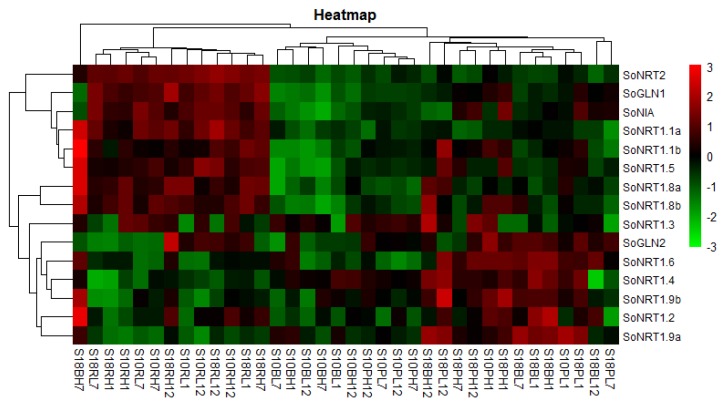
Expression profiles of *NRTs* genes in tissues of two spinach varieties under two nitrate concentration treatments. S10, the spinach So10 variety; S18, the spinach So18 variety; B, leaf blade; P, petiole; R, root; L, low nitrate (0.5 mmol·L^−1^) treatment; H, high nitrate (15 mmol·L^−1^) treatment; 1, 1 h; 7, 7 h; and 12, 12 h.

**Figure 6:**
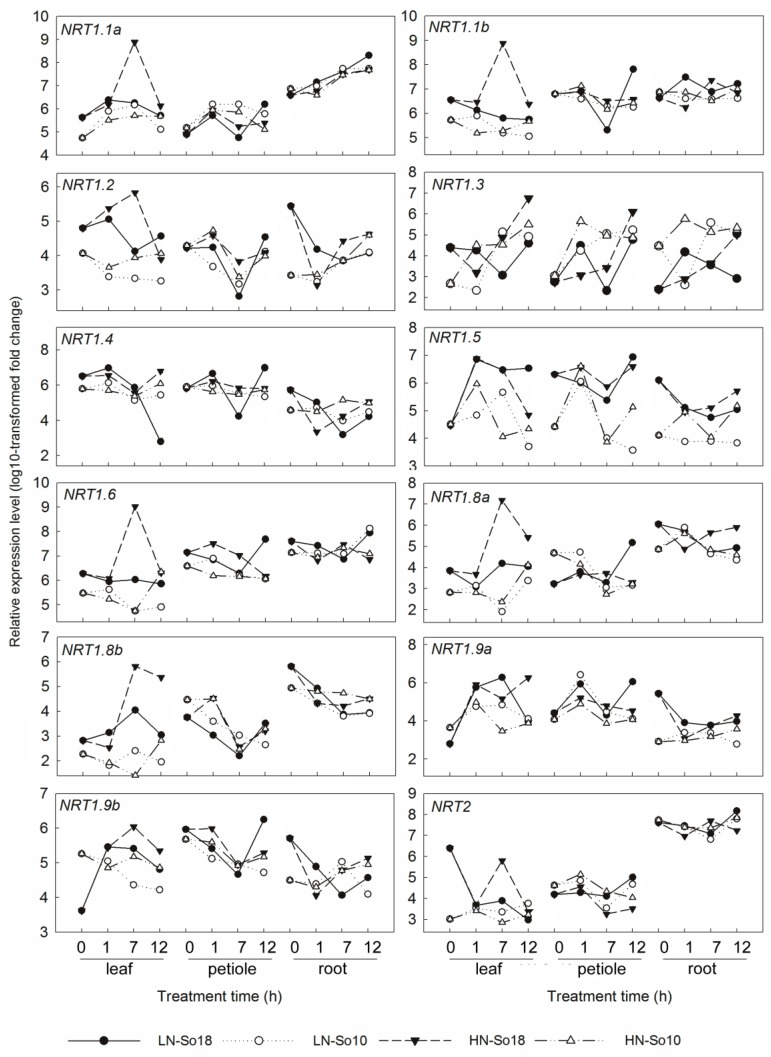
Expression analysis of genes encoding nitrate transporters in leaf blades, petioles and roots of two spinach varieties under two concentrations (0.5 and 15 mmol·L^−1^) of nitrate treatments by qRT-PCR. All values represent the averages of three replicates (see Supplementary Table S1 for details). LN-So18, So18 treated with low nitrate concentration; LN-So10, So10 treated with low nitrate concentration; HN-So18, So18 treated with high nitrate concentration; and HN-So10, So10 treated with high nitrate concentration.

**Figure 7:**
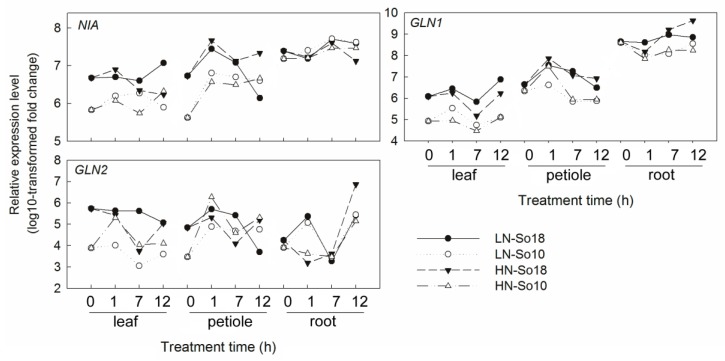
Expression analysis of nitrogen assimilation genes in leaf blades, petioles and roots of two spinach varieties under two concentrations (0.5 and 15 mmol·L^−1^) of nitrate treatments by qRT-PCR. All values represent the averages of three replicates (see Supplementary Table S1 for details). LN-So18, So18 treated with low nitrate concentration; LN-So10, So10 treated with low nitrate concentration; HN-So18, So18 treated with high nitrate concentration; and HN-So10, So10 treated with high nitrate concentration.

The tested genes displayed distinct expression patterns among different nitrate concentrations, tissues, treatment times and varieties (Figure 5, Figure 6 and Figure 7). Most of the tested gene transcripts were induced by high nitrate although their increases depended on treatment times and varieties (Figure S1). The relative expression levels of *SoNRT1.1a*, *SoNRT1.1b*, *SoNRT1.3*, *SoNRT1.4*, *SoNRT1.6*, *SoNRT1.8a*, *SoNRT1.8b* and *SoNRT1.9a* were at most 100–1000 times greater in varieties under high nitrate conditions than those under low nitrate conditions. *SoNRT1.1a*, *SoNRT1.1b*, *SoNRT1.6*, *SoNRT1.8a*, *SoNRT1.8b*, *SoNRT2*, *SoNIA* and *SoGLN1* were mainly expressed in roots, while *SoNRT1.4*, *SoNRT1.5*, *SoNRT1.9a*, *SoNRT1.9b* and *SoGLN2* were more abundant in leaf blades and petioles compared to roots (Figure 5). The expression level of all the tested genes varied along with the treatment time (Figure 6 and Figure 7). 

As shown by repeated measures analysis of variance, significant differences in gene expression were found between two spinach varieties (Table 1). Under low nitrate treatment conditions, the transcripts of all genes in leaf blades except *SoNRT* 1.4 of So18 were generally greater than those of So10 across all time points. The expression levels of *SoNRT1.1b*, *SoNRT1.4*, *SoNRT*1.5, *SoNRT1.6*, *SoNRT*1.9a, *SoNIA, SoGLN1* and *SoGLN2* in petioles and *SoNRT1.1b, SoNRT1.2, SoNRT1.4, SoNRT1.5, SoNRT1.8a, SoNRT1.8b, SoNRT1.9a* and *SoGLN1* in roots was greater in low nitrate treated So18 than those of So10. However, a significant increase of expression of five genes in petioles (*SoNRT1.1a, SoNRT1.3, SoNRT1.8a, SoNRT1.8b* and *SoNRT1.9b*) and one gene in root (*SoNRT1.3*) was found in So10, as there was a greater transcription level compared to those of So18. Under high nitrate treatment conditions, all of the tested genes showed higher expression levels in leaf blades of So18 than So10. The transcription level of six genes in petioles (*SoNRT1.4*, *SoNRT1.5*, *SoNRT1.6*, *SoNRT1.8a*, *SoNRT1.9a*, *SoNIA* and *SoGLN1*) and six genes in roots (*SoNRT1.2*, *SoNRT1.5*, *SoNRT1.8a*, *SoNRT1.9a*, *SoGLN1* and So*GLN2*) was increased in So18 than those in So10. In contrast, five genes in petioles (*SoNRT1.1a*, *SoNRT1.3*, *SoNRT1.8a*, *SoNRT1.9b* and *SoNRT2*) and three genes in roots (*SoNRT*1.3, *SoNRT1.9b* and *SoNRT2*) had higher expression levels in So10 than in So18. Other genes showed no significant differences in expression level between two varieties.

To understand the gene expression and their impact on plant nitrate content, we analyzed the correlations between nitrate contents and gene expression levels using Pearson correlation statistics. As shown by Table 2, the root nitrate content was positively correlated with the expression levels of *SoNRT1.2* (r = 0.84, *p* < 0.05) and *SoNRT1.8* (r = 0.82, *p* < 0.05) when under low nitrate treatment conditions. Under high nitrate treatment conditions, significant correlations were only observed in petioles. The petiole nitrate contents were positively correlated to the expression levels of *SoNRT1.4* (r = 0.84, *p* < 0.05) and *SoNRT*1.5 (r = 0.81, *p* < 0.05), but negatively correlated to *SoNRT1.3* (r = −0.93, *p* < 0.01).

## 3. Discussion

The main factors that influence nitrate content can differ in different plant species, varieties or tissues. To investigate the mechanism of nitrate accumulation in spinach, we analyzed the physiological and molecular characteristics of nitrate transport and assimilation. Our results showed that the plant nitrate contents differed between two spinach varieties, with higher nitrate contents in S18 and lower contents in S10, which occurred irrespectively of applied nitrate concentration. The differences in nitrate content of plants may be due to their differential capacities to absorb, reduce and assimilate nitrate. The fact that the nitrate uptake far exceeds its assimilation may account for the higher nitrate content in some cultivars [42]. In this study, the ^15^NO_3_^−^-N uptake rates and ^15^NO_3_^−^-N uptake amount of S18 were significantly greater than those of S10, while the NR and GS activities of So18 were similar to (NR activities among three tissues and leaf blade GS activities) or sometimes even lower than those of So10 (GS activities in petioles and roots). These data suggest that the nitrate accumulation differences between two tested spinach varieties is mainly due to their differential capacities to uptake and transport nitrate from the root to shoot.

Considering that nitrate uptake and translocation played a significantly more prominent role in nitrate accumulation in spinach, research on *SoNRTs* at the transcription level will help us to better understand the pattern of nitrate absorption in spinach and will be of great significance in further studies focusing on reducing nitrate accumulation in spinach. qRT-PCR analysis showed that the mRNA levels of the most *SoNRTs* in the high nitrate genotype So18 were greater than those in low nitrate variety So10, which is consistent with the greater nitrate uptake rates found in So18 compared to So10. These differences in *NRT* level may result from genetic variance of NRT or its regulation, which accounts for the higher nitrate uptake. A high nitrate uptake often requires high nitrate transportation and thus, we proposed that the higher *NRT* expression in So18 may have also indicated higher NO_3_^−^ transportation in So18. Thus, these differentially expressed *SoNRTs* genes may be involved in nitrate uptake or nitrate translocation in spinach, which provided candidate gene resources for further investigating nitrate accumulation in spinach.

Since nitrate was mainly accumulated in petioles, we paid more attention to the differentially expressed genes in petioles. Among them, four differentially expressed genes, *SoNRT1.4*, *SoNRT1.5*, *SoNRT1.6* and *SoNRT1.9a* were found to be more abundant in the leaf petioles of So18 than those of So10. In *Arabidopsis*, *NRT1.4* is expressed predominantly in the shoots and roots, which has been shown to be involved in nitrate storage in the leaf petiole [23]. The mutation of *AtNRT1.4* gene could greatly reduce petiole nitrate content [23]. In spinach, the homologous gene of *AtNRT1.4*, *SoNRT1.4* showed an *AtNRT1.4*-like tissue expression pattern, and its mRNA level was significantly increased under high nitrate provision. This suggests that *SoNRT1.4* may be a putative functional homolog of *AtNRT1.4*. Moreover, our study revealed a strong positive correlation of *SoNRT1.4* gene expression with nitrate content in petioles. This suggested that *SoNRT1.4* expression in petioles plays an important role in controlling the dynamic level of nitrate in petioles, which is a potential target or reducing nitrate accumulation. 

Similar to *SoNRT1.4*, *SoNRT1.5* expression level was also positively correlated to nitrate content in petioles. In Arabidopsis, AtNRT1.5 mediates the xylem-loading of nitrate and enhances the root-to-shoot transport of nitrate [15,43]. Similar to the expression pattern of its *Arabidopsis* homolog, *SoNRT1.5* mRNA level was also greatly increased by nitrate provision. We suggested that *SoNRT1.5* encoded protein has a similar function to AtNRT1.5 in enhancing shoot-to-root transport, and contributes to the greater nitrate accumulation in shoots of the So18 variety. Thus, *SoNRT1.5* may also have a potential to be used for reducing nitrate accumulation. However, *SoNRT1.5* expression was also strongly correlated with nitrate content in low nitrate treated roots. Sufficient nitrate is important for plant growth, especially when only a low concentration of nitrate available. Thus, we should consider whether the regulation of *SoNRTs* could reduce plant nitrate content while simultaneously not greatly affect plant growth. 

As for *SoNRT1.6*, its *Arabidopsis* ortholog *AtNRT1.7* was mainly expressed in the phloem tissue of older leaves, which mediates the phloem loading of nitrate in source leaves to remobilize nitrate from older leaves to nitrogen-demanding tissues [44]. In contrast, its ortholog *AtNRT1.6* was only expressed in the vascular bundles of the siliques and the funiculi, which are involved in delivering nitrate to developing seeds [45]. It seems that the *SoNRT1.6* encoded protein may fulfill similar physiological functions as its *Arabidopsis* homolog *AtNRT1.7* but not *AtNRT1.6*. However, in contrast to *AtNRT1.7* which mainly expressed in the shoot, the spinach *SoNRT1.6* showed reasonable expression in both the shoot and root. If the orthologous genes of spinach *NRT1.6* are also expressed in the phloem, the function of *SoNRT1.6* in nitrate loading and/or unloading to or from the shoot/root is possible. *SoNRT1.9a*, which is another highly expressed *SoNRT* gene in So18, was preferentially expressed in the shoots and is strongly induced by a high concentration of nitrate. In contrast, *AtNRT1.9* is expressed predominantly in roots where it is probably involved in the loading of nitrate into the root phloem to enhance downward nitrate transport in roots [43]. In addition, *AtNRT1.9* expression is not rapidly induced by nitrate [46]. This suggests that the *SoNRT1.9a* encoded protein may fulfill different physiological functions compared to its *Arabidopsis* homolog. The different orthologous NRT1.9 expression in relation to tissue specificity and N-supply have also been found in other plant species. The *Cucumis sativus* orthologous *NRT1.9* gene expression is highly up-regulated by high nitrate supply in all vegetative organs except for roots [22]. While none of the wheat homologous *TaNRT1.9* genes showed dominance for root expression [47]. This emphasizes the complexity and variation in gene regulation in relation to nutrient uptake in crop plants in comparison to model plant. Being different with *SoNRT1.4* and *SoNRT1.5*, both *SoNRT1.6* and *SoNRT1.9a* expression showed no statistically significant correlation with the petiole nitrate content. Considering their strongly increased transcripts under high nitrate, their detailed roles in nitrate response still need to be further investigated.

Four *SoNRTs* (*SoNRT1.1a*, *SoNRT1.3*, *SoNRT1.8a* and *SoNRT1.9b*) have high levels of expression in the petioles of So10. Even though these *SoNRTs* might be involved in nitrate accumulation, their contributions to nitrate accumulation in plant might be not as high as their contributions in So18, especially considering that more *SoNRTs* were abundant in So18 than in So10. Interestingly, among these genes, *SoNRT1.3* expression was negatively correlated with the nitrate content in petioles, which indicated that the *SoNRT1.3* encoded protein may negatively regulate the dynamic level of nitrate in petioles. As far as we know, the functional role of its *Arabidopsis* homolog, AtNRT1.3, remains unclear although it has been proposed that it has a possible role in polyamine resistance [48]. We proposed that *SoNRT1.3* encoded protein fulfills different physiological function from AtNRT1.3 and may play an important role in reducing nitrate accumulation in spinach.

It should be noted that the expression patterns of these *SoNRTs* in this study are mainly related to an early nitrate response (0–12 h), considering the *NRT* expression is spatiotemporally regulated and may change with short- and long-term nitrate treatment [49,50]). Moreover, allelic differences and/or different gene regulation efficiencies between varieties may also affect NRT expression [51] and thus, the screening of these *SoNRT* genes is only the first step in creating a comprehensive understanding of the nitrate accumulation mechanisms in spinach. More functional analyses of these candidate *SoNRT*s in nitrate accumulation in spinach need to be conducted, especially under long-term nitrate treatment conditions.

As for the nitrate assimilation related genes, it seems suggest that the spinach *NRT* expression is compatible with the expression of *NIA* and *GLNs*, for their expression levels were all higher in So18 than in So10. It was reported that initial exposure to nitrate causes an increase of NR activity and NR-mRNA, while the NR induction is inhibited by GS and/or its reduced N-compounds (e.g., ammonium or amino acids) after long time exposition [50]. Similar to NR, a rapid and transient increase in GS activity was also found under nitrate treatment [52]. Based on these findings, we suppose that the higher nitrate uptake and higher *NRT* expression in So18 cause a higher level of nitrate content in some cells, and thus stimulate an increased nitrate reduction, meaning increasing *NIA* and *GLNs* expression in So18 under early nitrate treatment conditions. With the prolonging of nitrate treatment, and the long-distance transport of N, the nitrate content varies in different tissues and subcellular fractions, which may accounts for the dynamic regulation of NR and GS by nitrate supply. Meanwhile, the net nitrate uptake may also be inhibited due to a reduced demand for nitrate or feed-back control by various C/N metabolites, which further regulated the *NRT* expression and also the nitrate assimilation related genes expression. The interaction between *NRT*, *NIA* and *GLNs* in response to nitrate condition and their roles in nitrate accumulation are promising targets for further research. During the experiment period, despite the relatively higher *SoNIA* and *SoGLNs* transcription levels in So18, the abundances of *SoNRTs* increased greatly compared to that of *SoNIA* and *SoGLNs* in response to a high nitrate concentration. As a result, the *SoNIA* and *SoGLNs* abundances were not high compared to the super-high nitrate concentration in So18.

It should be note that, the expression patterns of *SoNIA*, *SoGLN1* and *SoGLN2* in response to nitrate provision were not consistent with changes in their enzyme activities. Generally, the mRNA levels of these genes were higher in So18 than in So10 irrespectively of nitrate concentration, while NR activities did not differ between two varieties and GS activities fluctuated greatly between all treatments. The absence of consistency between the transcripts and enzyme activities may be due to the post-transcriptional and post-translational regulation, while the highly regulated NR enzyme not only can be regulated by many internal (e.g., internal nitrate content, reduced N-compounds) and external (e.g., nitrate application, light, CO_2_) factors [53,54,55,56]. Another explanation for this phenomenon may be related to the study is mainly for early expression response of homologous genes, while the enzyme activity tested here is a mixture of the homologous products. The detailed roles of *NIA* and *GLNs* in nitrate accumulation still need to be further investigated.

## 4. Materials and Methods 

### 4.1. Plant Sample Preparation

Spinach seeds (So10 and So18) were sterilized in 70% ethanol for 5 min and 10% (*v*/*v*) H_2_O_2_ for 30 min. After this, they were rinsed with distilled water, and germinated in a plant growth chamber under a 10 h day/14 h night cycle (23 °C /16 °C day/night temperature cycle) with 60–70% relative humidity. The uniform seedlings with four leaves were transferred into a nutrition solution. The nutrient solution which was used to support plant growth contained: 0.2 g·L^−1^ KNO_3_, NaH_2_PO_4_ 2H_2_O 0.09 g·L^−1^, Na_2_HPO_4_ 12H_2_O 0.15 g·L^−1^, CaCl_2_ 2H_2_O 0.29 g·L^−1^, MgSO_4_ 7H_2_O 0.49 g·L^−1^, NaFeEDTA 0.02 g L^−1^, H_3_BO_3_ 2.86 mg·L^−1^, MnCl_2_ 4H_2_O 1.81 mg·L^−1^, ZnSO_4_ 7H_2_O 0.22 mg·L^−1^, CuSO_4_ 5H_2_O 0.08 mg·L^−1^ and NaMoO_3_ 2H_2_O 0.09 mg·L^−1^. The pH was adjusted using 0.1 mol·L^−1^ NaOH and 0.1 mol·L^−1^ HCl to be within a pH range of 5.0–7.0. After two weeks, seedlings of uniform size and growth were picked randomly and transferred to −N (N free) solution for 4 d (other nutrients remained as before), before being immersed in 0.5 mmol·L^−1^ KNO_3_ or 15 mmol·L^−1^ KNO_3_ (other nutrients remained as before) from 0 to 12 h. The high nitrate concentration (15 mmol·L^−1^) represents the maximum amount of nitrate for hydroponic spinach growth. Our pre-experiment indicated that the tested spinach varieties could grow well under 15 mmol·L^−1^ nitrate when compared with 20 and 30 mmol·L^−1^ concentrations (data not shown). Plants were harvested and three independent replicates (three plants for each replicate) were collected for each sample. Plant materials were frozen in liquid nitrogen immediately and stored at −80 °C until subsequent analyses.

### 4.2. ^15^NO_3_^−^-N Uptake Rate

Nitrate uptake was determined by the ^15^N isotopic tracer method as previously described [57]. Briefly, the plants were exposed to 0.1 mmol·L^−1^ CaSO_4_ for 1 min, before being immersed in a complete nutrient solution containing 0.5 or 15 mmol·L^−1 15^NO_3_^−^ for 0 h, 1 h, 4 h, 7 h or 12 h. After this, they were rinsed in 0.1 mmol·L^−1^ CaSO_4_ for 1 min before washing with deionized water. The plant shoots and roots were freeze-dried, weighed to determine the dry weight, and subsequently milled to fine powder. The total contents of N and ^15^N were determined using a Vario ELIII elemental analyzer (Elementar, Langenselbold, Germany). The rates of ^15^N uptake were calculated by dividing total plant ^15^N amount at time by the root dry weight in grams (µg N·g^−1^ root DW·h^−1^). The net ^15^N uptake amount by plant was represented as ^15^NO_3_^−^-N excess (µg N·g^-1^ plant DW).

### 4.3. Measurements of Nitrate Content and Enzymes Activity

Plant samples were sampled and washed with flowing water to obtain the whole plants. They were then divided into roots, petioles, and leaf blades to determine the nitrate contents in different organs. Nitrate content determination followed the method described by Tang et al. [58]. The method of determining NR and GS activity was described by Tang et al. [58] and Wang et al. [59]. One unit of NR activity is defined as the production of 1 µg nitrite per hour per fresh weight (μg NO_2_^−^·g^−1^ FW·h^−1^). One unit of GS activity is defined as 1 A_540_·g^−1^ FW·h^−1^.

### 4.4. Identification of Nitrogen-Responsive Genes and Gene Expression Analysis

The Blast software was used to search the spinach genes against the spinach genomics database (http://www.spinachbase.org/cgi-bin/spinach/index.cgi) [60] and National Center for Biotechnology Information (NCBI) with the corresponding 10 functionally characterized *Arabidopsis* gene sequences (*AtNRT1.1*, *AtNRT1.2*, *AtNRT1.3*, *AtNRT1.4*, *AtNRT1.5*, *AtNRT1.6*, *AtNRT1.7*, *AtNRT1.8*, *AtNRT1.9* and *AtNRT2.1*) as the initial query. The information of the identified genes is listed in Table 3. To improve the quality of primers and prevent mismatch and DNA contamination, the specific primers were obtained from the non-conserved regions and span an exon-exon junction. Gene specific primers designed by Prime 5 are listed in Table 4.

Total RNA was extracted using TRIzol reagent (Invitrogen, Carlsbad, CA, USA) according to the manufacturer’s instructions. Two μg of DNase-treated RNA was used to synthesize cDNA using the PrimeScript™ RT Master Mix (TAKARA, Tokyo, Japan). qRT-PCR was performed using a SYBR^®^ Premix Ex Taq™ II (TAKARA, Tokyo, Japan) on a ABI 7500 Real-Time PCR System (Applied Biosystems, Foster, CA, USA). The initial denaturing time was 10 min, which was followed by 35 PCR cycles consisting of 94 °C for 0 s, 63 °C for 5 s and 72 °C for 10 s. A melting curve was run after the PCR cycles, which was followed by a cooling step. The relative quantitation of gene expression of qRT-PCR was measured via the 2^−ΔΔCt^ method [61], with *18s rRNA* used as the endogenous reference gene. Twice the ^Δ^CT value of *SoNRT1.1a* in leaves of So10 under low nitrate condition was used as the control group. Data were presented as log10 transformation.

### 4.5. Statistical Analysis

Significant differences of physiological indices between different samples were calculated using a multiple factor analysis of variance with a LSD test (at a significance level of *p* < 0.05). Repeated measures analysis of variance was used to test for the effects of spinach genotype on gene expression (*p* < 0.05). The data are expressed as the mean and the standard deviation (mean ± SD, *n* = 3). The relationship between nitrate content and gene expression was determined using the Pearson’s correlation analysis. Statistical calculations were performed using SPSS software version 13.0 (IBM, New York, NY, USA).

## 5. Conclusions

The nitrate accumulation differences between two tested spinach genotypes may be due to their differential capacities to uptake and transport nitrate from the root to shoot. The highly expressed So*NRT* genes found in So18, especially *SoNRT1.4* and *SoNRT1.5*, may facilitate nitrate transport to petioles and contribute to excessive nitrate accumulation in So18. The *SoNRT1.3* seems to play a negative role in shoot nitrate accumulation and thus, may be a potential target for reducing spinach nitrate content. Other *SoNRTs*, which may fulfill different physiological functions from their *Arabidopsis* homologs, may also be involved in the early nitrate response in spinach, although more evidence is needed to prove their roles in nitrate accumulation. The identification of these *SoNRT* genes is only the first step in providing comprehensive insights into nitrate accumulation mechanisms in spinach. As a first attempt, the present analysis will be useful for further investigations for understanding the roles of *SoNRTs* in nitrate accumulation in spinach.

## Figures and Tables

**Table 1:** Results of repeated measures ANOVA for the effects of varieties on gene expression.

Nitrate Concentration Treatment	Gene Symbol	Leaf blade		Petiole		Root	
		*F* Value	*P* Value	*F* Value	*P* Value	*F* Value	*P* Value
0.5 mmol L^−^^1^	*SoNRT1.1a*	65.03	0.001	*70.66*	*0.001*	1.172	0.340
	*SoNRT1.1b*	165.68	0.000	17.38	0.014	32.42	0.005
	*SoNRT1.2*	218.12	0.000	4.42	0.103	96.52	0.001
	*SoNRT1.3*	34.63	0.004	*676.55*	*0.000*	*345.14*	*0.000*
	*SoNRT1.4*	1.96	0.235	35.10	0.004	1.43	0.298
	*SoNRT 1.* *5*	308.94	0.000	529.18	0.000	305.17	0.000
	*SoNRT1.6*	1389.78	0.000	182.38	0.000	1.87	0.243
	*SoNRT1.* *8a*	393.56	0.000	0.17	0.702	25.67	0.007
	*SoNRT1.* *8b*	291.00	0.000	*175.47*	*0.000*	38.39	0.003
	*SoNRT1.9a*	18.23	0.013	296.94	0.000	176.73	0.000
	*SoNRT1.9b*	75.74	0.001	*96.22*	*0.001*	10.19	0.033
	*SoNRT2*	1097.97	0.000	0.26	0.637	7.44	0.053
	*SoNIA*	182.52	0.000	71.58	0.001	0.03	0.879
	*SoGLN1*	526.29	0.000	484.55	0.000	153.67	0.000
	*SoGLN2*	1479.40	0.000	213.74	0.000	2.07	0.223
15 mmol·L^−^^1^	*SoNRT1.1a*	1684.78	0.000	*40.11*	*0.00*	0.09	0.775
	*SoNRT1.1b*	915.06	0.000	0.99	0.375	1.58	0.277
	*SoNRT1.2*	351.99	0.000	1.33	0.313	69.87	0.001
	*SoNRT1.3*	166.19	0.000	*190.68*	*0.000*	*396.17*	*0.000*
	*SoNRT1.4*	191.83	0.000	39.81	0.003	7.76	0.050
	*SoNRT 1.* *5*	4024.10	0.000	255.51	0.000	357.70	0.000
	*SoNRT1.6*	2276.30	0.000	267.66	0.000	1.09	0.356
	*SoNRT1.* *8a*	3253.03	0.000	*9.88*	*0.035*	88.67	0.001
	*SoNRT1.* *8b*	1447.21	0.000	7.51	0.052	0.23	0.657
	*SoNRT1.9a*	210.99	0.000	106.84	0.000	85.43	0.001
	*SoNRT1.9b*	33.88	0.004	*16.43*	*0.015*	*156.65*	*0.000*
	*SoNRT2*	1431.02	0.000	*136.43*	*0.000*	*27.52*	*0.006*
	*SoNIA*	452.08	0.000	219.11	0.000	0.01	0.921
	*SoGLN1*	493.49	0.000	78.36	0.001	475.91	0.000
	*SoGLN2*	556.74	0.000	0.65	0.467	3329.69	0.000

The statistically significant values in italics and regular font indicate that the corresponding transcripts were significantly more abundant in So10 and So18, respectively. All results were considered significant at the *p* < 0.05 level, *n* = 3.

**Table 2:** Pearson correlation between nitrate contents and gene expressions.

	Low Nitrate Treatment			High Nitrate Treatment		
	Leaf Blade	Petiole	Root	Leaf Blade	Petiole	Root
*SoNRT1.1a*	−0.24	−0.40	0.51	0.53	−0.10	−0.49
*SoNRT1.1b*	0.40	0.01	0.56	0.53	0.00	−0.51
*SoNRT1.2*	0.39	0.06	0.84 *	0.78	0.08	−0.69
*SoNRT1.3*	0.00	−0.15	0.33	−0.75	−0.93 **	−0.54
*SoNRT1.4*	0.06	−0.05	0.43	−0.07	0.84 *	−0.51
*SoNRT1.5*	0.29	0.00	0.82 *	0.28	0.91 *	0.17
*SoNRT1.8a*	0.24	−0.29	−0.21	0.13	0.11	0.06
*SoNRT1.8b*	−0.29	−0.47	0.17	0.07	0.05	−0.05
*SoNRT1.6*	−0.09	−0.03	0.39	0.79	0.21	−0.59
*SoNRT1.9a*	−0.28	−0.38	0.05	0.42	0.47	−0.51
*SoNRT1.9b*	0.19	0.20	0.32	0.69	0.28	−0.59
*SoNRT2*	−0.11	−0.26	0.61	0.56	−0.32	−0.62
*SoNIA*	0.10	−0.02	−0.21	0.47	0.76	0.02
*SoGLN1*	0.51	0.18	0.36	0.26	0.37	−0.17
*SoGLN2*	0.29	−0.08	0.58	0.16	−0.45	−0.77

* indicates correlation is significant at the 0.05 level; ** indicates correlation is significant at the 0.01 level.

**Table 3:** Genes involved in nitrate transport and assimilation identified in spinach.

Gene	Ortholog Locus	Spinach Source Gene	Spinach Gene Symbol	Position of Predicted Genes	Length of ORF	Length of Protein
*AtNRT1.1*	AT1G12110.1	Spo26413	*SoNRT1.1a*	24047-30641	1782	593 aa
	AT1G12110.1	Spo11551	*SoNRT1.1b*	27689-32901	1230	409 aa
*AtNRT1.2*	AT1G69850.1	Spo24381	*SoNRT1.2*	9278773-9283346	1767	588 aa
*AtNRT1.3*	AT3G21670.1	Spo18484	*SoNRT1.3*	32544817-32547940	1737	578 aa
*AtNRT1.4*	AT2G26690.1	Spo05548	*SoNRT1.4*	370979-375454	1755	584 aa
*AtNRT1.* *5*	AT4G21680.1	Spo23405	*SoNRT 1.8*	43590162-43598583	1800	599 aa
*AtNRT1.6* */1.7* ^1^	AT1G27080.1	Spo25744	*SoNRT1.6*	39077918-39086543	1875	624 aa
*AtNRT1.* *8*	AT1G32450.1	Spo00021	*SoNRT1.5a*	111335-115169	1752	583 aa
	AT1G32450.1	Spo00022	*SoNRT1.5b*	135693-137789	1773	590 aa
*AtNRT1.9*	AT1G18880.1	Spo20247	*SoNRT1.9a*	9835695-9840007	1764	587 aa
	AT1G18880.1	Spo20248	*SoNRT1.9b*	9830221-9833091	1776	591 aa
*AtNRT2.1*	AT1G08090	Spo09966	*SoNRT2*	44925216-44928954	1584	527 aa
*AtNIA*	AT1G77760	Spo23607	*SoNIA*	51343436-51348754	867	288 aa
*AtGLN1*	AT5G37600	Spo17102	*SoGLN1*	9174181-9181171	1785	594 aa
*AtGLN2*	AT5G35630	Spo06798	*SoGLN2*	19807870-19834770	2664	887 aa

^1^ Spo25744 is the ortholog of two *AtNRT*s (*AtNRT1.6* and *AtNRT1.7*).

**Table 4:** Specific primers for *NRT*, *NIA* and *GLN* in spinach.

Symbol	Gene ID	Primer Sequence (5′→3′)	
		Forward	Reverse
*SoNRT1.1a*	Spo26413	TAAGACTGGCGGTTGG	GACAGAGCATGAAGGAGG
*SoNRT1.1b*	Spo11551	GTGAGGCATGTGAAAGATTA	CCACCAAGTAGGCAGAGC
*SoNRT1.2*	Spo24381	TTGTGGAGGTGCTGGAGA	ACGGCTGATTTAGATGGTGAGA
*SoNRT1.3*	Spo18484	TTTGGAGGATTTGTCG	ATGTAAAGAGCAGCGTAT
*SoNRT1.4*	Spo05548	GATAGCCGTGAACCTTG	GCGAAAATTGCGACA
*SoNRT1.* *5*	Spo23405	CAATCAGGAAATGGAGGAC	TGTTATGGGACAAAGACG
*SoNRT1.6*	Spo25744	CCGTTTCCTCGTTCCAGC	ACCCTTCAGCAAGCCCTA
*SoNRT1.* *8* *a*	Spo00021	CATCTATCGCCGTGTT	GCTTAGACTACTTGCCTCT
*SoNRT1.* *8* *b*	Spo00022	TTTTCGGCGTAGGAGT	TGTGGCGATGTTTGGT
*SoNRT1. 9a*	Spo20247	GGAGTCCCCTTACGAGTG	TCCTTCTGGGTTTATTTTG
*SoNRT1.9b*	Spo20248	GTGAGTTGGAGCATCGG	CGTCAGGGGTTATTATCG
*SoNRT2*	Spo09966	TTTGCCTTCTCGGTCTC	CGCCGCGAATGTGGATAT
*SoNIA*	Spo23607	CTTGCCAATTCTGAAGCT	TAGTCCAGGCGTTGATAG
*SoGLN1*	Spo17102	GGATATTTCGAGGACAGG	TGGTTTCCAAAGAAGGGT
*SoGLN2*	Spo06798	CGGAGAAGGGAATGAAA	CACGGATTGAGCAACCAC
*18SrRNA*	Spo24625	CCATAAACGATGCCGACCAG	AGCCTTGCGACCATACTCCC

## Supplementary Materials

The following are available online. Table S1: The relative expression levels of nitrate accumulation related genes under 0.5 and 15 mmol·L^−1^ nitrate treatment conditions; Figure S1: The ratios of the relative gene expression under high to low nitrate treatments.
