# Supplementary material for: Nitrate Accumulation and Expression Patterns of Genes Involved in Nitrate Transport and Assimilation in Spinach
Source: Molecules. 2018 Sep 2;23(9):2231. doi: 10.3390/molecules23092231 (PMC6225323; doi:10.3390/molecules23092231)
Supplement: Supplementary file 1 [file molecules-23-02231-s001.zip › Figure S1.pdf]

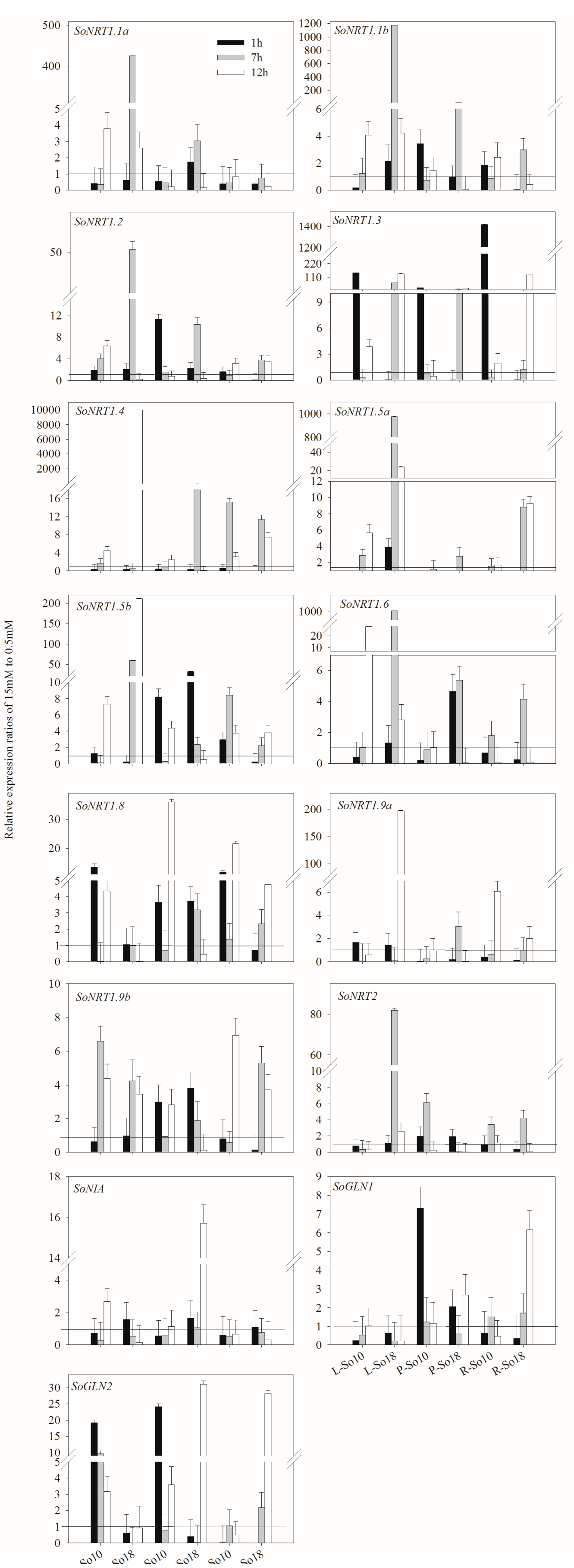

Figure S1: The ratios of the relative gene expression under high to low nitrate treatment conditions. L-So10, the leaf blades of So10; L-So18, the leaf blades of So18; P-So10, the petioles of So10; P-So18, the petioles of So18; R-So10, the roots of So10; and R-So18, the roots of So18.
